# Preface to special topic on information and intelligent metasurfaces

**DOI:** 10.1093/nsr/nwad197

**Published:** 2023-07-20

**Authors:** Tie Jun Cui, Marco Di Renzo

**Affiliations:** Southeast University, China; Université Paris-Saclay, France

In the past decades, information science and technology has developed rapidly, but has also encountered some bottlenecks. Its further development requires some original driving force of underlying physics (such as quantum mechanics, metamaterial physics, etc.). On the other hand, physics development urgently requires breakthrough applications in the field of information. Therefore, deep integration of physics and information science is of great significance to physical science and new information technologies. Information metamaterial is a representative of deep fusion of electromagnetic physics and digital information. The original proposal of metamaterial is to seek unusual media (or effective material) parameters (e.g. negative permittivity and negative permeability) and find anomalous phenomena (e.g. negative refraction and perfect imaging) that do not exist in nature using periodic and/or aperiodic arrays of meta-atoms in the subwavelength scale. Hence studies of metamaterials and metasurfaces have long been focused on the physical perspective. In 2014, digital coding and programmable metamaterials and metasurfaces were proposed to control electromagnetic fields in a reprogrammable way by digitally defining these meta-atoms, opening a new era of studying the metamaterials from the information perspective, and resulting in the appearance of information metamaterials. Information metamaterials and metasurfaces can not only manipulate the electromagnetic waves, but also directly modulate the digital information, and hence fuse together the electromagnetic physics and digital information.

On the other hand, electronic and information technology, together with industry, needs new physics to satisfy challenging requirements for new-generation wireless communications. In 2019, a concept of reconfigurable intelligent surface (RIS) or intelligent reflecting surface (IRS) was proposed independently by several groups in the wireless communication community to control the wireless environment as desired and enhance wireless coverage. This virtual RIS should have the capability to smartly reflect and scatter the electromagnetic waves and beams to the required directions and regions and generate the appropriate waveforms for wide coverage in real time. The requirements of RIS for wireless communications can be entirely fulfilled by the programmable metasurface in the electromagnetic community, and hence a realistic RIS platform was built up using the metasurface. Nowadays, with close collaboration between the electromagnetic physics and wireless communication societies, information metamaterials and RISs have made great progresses both independently and jointly, and have become one of the potential key techniques for sixth-generation (6 G) wireless communications under the framework of IMT2030.

In the above context, we invited eight leading scientists in the electromagnetic and wireless communication fields from across the world to publish this special topic on information and intelligent metasurfaces. The special topic includes one research article, two review articles, four perspectives articles, one research highlight, and one interview. In the research highlight, Vincenzo Galdi introduces a space- and frequency-division multiplexing wireless architecture without the need for complex and bulky radio-frequency components such as digital-to-analog converters and mixers [1]; while in the research article by Hanting Zhao and co-workers, an intelligent indoor metasurface robotics is proposed, in which all sensing, computing, and intelligent functions are performed in the information metasurface ‘brain’, while the robotic limb merely executes the instructions wirelessly received from the brain [2]. Both articles are based on the unique features of the information metasurfaces. In the four perspectives contributions, Ertugrul Basar provides a brief overview of RIS applications for emerging multiple-input multiple-output (MIMO) systems [3]; Xiaodan Shao and Rui Zhang present a new application scenario on target-mounted RIS to enhance wireless sensing [4]; Wei Wang and Wei Zhang discuss signal processing (channel estimation, beam training, and beamforming through learning) for RIS-assisted millimeter-wave and terahertz communications [5]; while Younes Ra’di *et al*. review and overview the metasurfaces for next-generation wireless communication systems [6]. In the two review articles, Yu Han *et al*. introduce recent advances on the acquisition of channel state information in RIS-assisted wireless communication systems from the perspectives of architecture improvement and mathematical solution [7]; while Vasileios Ataloglou *et al*. present some of the recently proposed passive and dynamic metasurfaces for enhancing the performance of wireless communication systems based on their controllability and programmability [8]. Finally, Linglong Dai assembles an interview with Professor Tie Jun Cui on the original thought, the current status, and the future trends of information metamaterials and metasurfaces [9].

In the past several years, there have been many advancements in the information metasurfaces and RIS fields, and this special topic only reflects some representative works. In fact, this is a rapidly developing area in both electromagnetic physics and wireless communications, which require new theories, new methods, new designs, and new technologies. We sincerely hope that the special topic will inspire scientists and engineers from multidisciplinary fields to explore this area and push forward industrial applications.
